# Impact of multi-channel follow-up as continuous nursing on cancer pain control, adverse reactions, and quality of life in patients with digestive tract tumors: a controlled study of 136 cases

**DOI:** 10.3389/fonc.2026.1868504

**Published:** 2026-07-20

**Authors:** Jue Fu, Jing Cheng, Chenxia Ju, Kejun Dai, Lei Wang

**Affiliations:** 1 Department of Tumor Radiotherapy, Changzhou Cancer Hospital, Jiangsu Province, Changzhou, Jiangsu, China; 2 Department of Nursing, Changzhou Cancer Hospital, Jiangsu Province, Changzhou, Jiangsu, China; 3 Medical Oncology, Changzhou Cancer Hospital, Jiangsu Province, Changzhou, Jiangsu, China

**Keywords:** cancer pain, constipation, continuous nursing, digestive tract tumors, quality of life (QoL)

## Abstract

**Objective:**

This study was designed to assess the effectiveness of multi-channel follow-up as a continuous nursing intervention in monitoring pain relief, adverse effects and quality of life in patients with digestive tract tumors following their discharge.

**Methods:**

A total of 136 patients were recruited from the Department of Medical Oncology and Department of Tumor Radiotherapy from July 2023 to July 2025 in this prospective controlled study. Participants were averagely and randomly assigned into the experimental group (n=68) that received multi-channel follow-up (including nurse-led telephone assessments, WeChat platform-based monitoring, and home visits) and a control group (n=68) that received standard telephone follow-up. Pain intensity and patient-reported satisfaction with pain control were primary outcomes. Secondary outcome measures included constipation, other analgesic-related adverse events, and quality of life.

**Results:**

The experimental group had statistically significant lower pain scores and higher pain control satisfaction compared with the control group. The incidence of constipation was also lower in the experimental group, while no remarkable difference was found in other analgesics-related adverse drug reactions (ADRs). The quality of life was significantly improved in the experimental group as the increased number of cases with good sleep quality (PSQI ≤7) was observed in the experimental group when compared to the control group. The association between pain intensity and sleep disturbance appeared to weaken after accounting for group assignment.

**Conclusion:**

Multi-channel follow-up as continuity-of-care nursing improved pain control, reduced constipation, and enhanced sleep quality among post-discharge patients with digestive tract tumors. The observed association between pain intensity and sleep quality was attenuated after adjustment for group assignment and should be interpreted as associative rather than causal.

## 1 Introduction

Pain remains a common and clinically significant symptom in patients with digestive tract tumors and might be related to physical activity, emotional distress, and general quality of life (QoL) impairment (1). A recent systematic review and meta-analysis reported that the overall prevalence of pain among patients with cancer was 44.5%, and moderate-to-severe pain was present in 30.6% of patients, indicating that cancer pain remains a major supportive-care issue despite improvements in assessment and management (2). In the patients with digestive tract tumors, pain was independently associated with poorer role functioning, greater emotional distress, and higher levels of depression (3, 4). The correlation between this disease and pain is a salient feature in this population, highlighting that pain is much more than a physical symptom (5). Although clinical practice guidelines from the European Society for Medical Oncology (ESMO) recommend that a large proportion of cancer pain can be adequately controlled with existing pharmacological and non-pharmacological interventions, inadequate pain management is a common occurrence: more than half of the patients in developed countries and even more in the developing world are said to be undertreated (6). This creates a significant challenge in translating evidence into clinical practice, and the challenge is especially evident in the post-discharge period. At this time care tends to be fragmented and follow-up insufficient and as a result both symptoms and emotional burden of cancer pain may be overlooked or exacerbated (7, 8).

Traditional, single-modality follow-up reveals care gaps post-discharge. Standard physician-led outpatient visits may not capture the dynamic course of cancer pain, while reactive, patient-initiated reporting can delay symptom management and increase emergency visits (9, 10). In response, multi-channel continuity-of-care approaches, including nurse-led remote follow-up, ongoing symptom monitoring, and individualized care navigation, have been proposed to improve continuity (11). Evidence supports nurse-led remote follow-up for reducing cancer pain severity and individualized follow-up for greater pain reduction than usual care (11, 12). Collectively, these findings support multi-channel models that sustain care post-discharge, enable timely adjustment of analgesic regimens, and address psychosocial needs often missed in routine follow-ups.

Although interest in continuity of care in cancer patients is increasing, significant uncertainties remain regarding the effectiveness and implementation of multi-channel continuity-of-care programs specifically for patients with digestive tract tumors. First, the studies on cancer pain management were mainly centered on pharmacologic treatment including opioid and non-opioid analgesics. There is limited data on how nursing-led follow-up models influence pain trajectories and treatment-related adverse reactions like constipation. Constipation is a common complication in patients with digestive tract tumors, with an incidence of 40–60% among those receiving analgesic therapy.

Second, although it is accepted from many studies that there is a negative association between pain and quality of life, it has not been widely investigated whether structured follow-up interventions could influence this relationship. This is especially true in terms of whether structured follow-up interventions could attenuate the association between pain and quality of life, which is particularly strong for those patients suffering from late-stage digestive tract tumors. Third, patients with digestive tract tumors have other special challenges, such as treatment-related gastrointestinal toxicities and quickly progressing disease. However, personalized follow-up strategies for this group of patients are not consolidated.

This controlled study aims to address these gaps by evaluating the impact of multi-channel follow-up as a continuous nursing strategy on 136 patients from the Department of Medical Oncology and Department of Tumor Radiotherapy. In order to evaluate the results of the experimental group receiving multi-channel follow-up against the control group receiving care as usual, we aim to realize the following distinct goals: (1) to assess the increment in pain control related outcomes using the NRS scores as well as the patient-reported pain control satisfaction; (2) to evaluate the variation of treatment-related adverse reactions with specific attention to constipation and side effects associated with analgesics; (3) to assess the increment of the quality of life using PSQI as the primary indicator; and (4) to analyze whether multi-channel intervention modify the relationship between pain severity and quality of life. Overall, this study contributes in the following ways. First, it assesses a scalable post-discharge care delivery model for patients with digestive tract tumors. Furthermore, it highlights the feasibility of nursing in continuity of care provision following hospital discharge. At the same time, this study provides disease-specific evidence in pain management and symptom control for this group.

## 2 Materials and methods

### 2.1 Study design and participants

This prospective study was conducted from July 2023 to July 2025 and was approved by the Institutional Review Board of Changzhou Tumor Hospital, approval number: 2025 (SR) No. 044. The study design complied with the Declaration of Helsinki, and the participants or their legal representatives provided written informed consent prior to enrollment.

#### 2.1.1 Eligibility criteria

**Figure 1:**
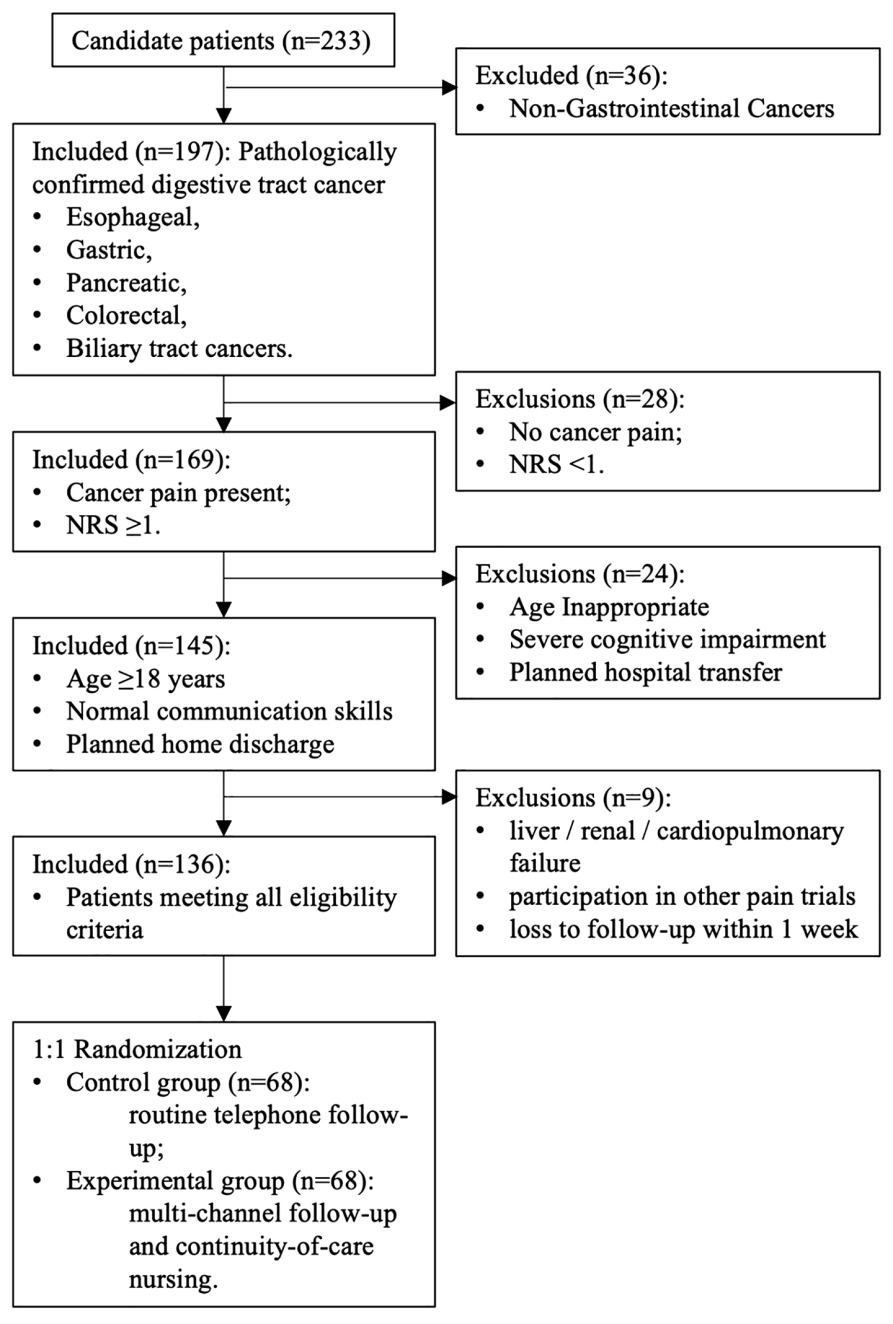
Flowchart of Patient screening, enrollment, and randomization.

As shown in **Figure 1**, inclusion criteria are as follows. First, participants had pathologically confirmed digestive tract tumors, including esophageal, gastric, pancreatic, colorectal, and biliary tract tumors. Second, they had cancer-related pain with a baseline NRS score ≥1. Third, they were aged ≥18 years. Fourth, they had the ability to communicate and complete symptom assessments, and planned to be discharged to home instead of transferred to other institutions. The included population were randomly allocated 1:1 to the experimental group (EG) or control group (CG) using a computer-generated random number sequence prepared by an independent researcher. Group assignments were placed in sealed opaque envelopes and opened sequentially after enrollment. The target sample size of 136 (68 per group) was determined based on feasibility and the expected patient flow within the study period.

Exclusion criteria included four aspects. First, participants had non-digestive tract tumors. Second, participants who had no cancer-related pain. Third, participants with inappropriate age, severe cognitive impairment and planned hospital transfer. Lastly, the patients who had liver, renal, and cardiopulmonary failure, in addition to those who participated in other pain trials and loss to follow-up within 1 week.

#### 2.1.2 Participant enrollment and grouping

A total of 136 eligible patients were consecutively enrolled and randomly assigned in a 1:1 ratio to the control group or experimental group using simple randomization. The control group consisted of 68 patients who had undergone routine post-discharge follow-up. This involved single-channel phone calls once a week for four consecutive weeks post-discharge to assess symptom status and medication adherence.

The experimental group had 68 patients and received multi-channel follow-up as continuous nursing. This included three parts. First, nurse-led telephone follow-up was conducted with the same frequency as the control group, and standardized symptom assessment was performed during the calls. Second, WeChat group management was conducted with daily medication reminders and a symptom log via an automated message, as well as a 24-hour service for emergency symptom questions. Third, a home visit by a designated nurse was made 2 weeks post-discharge for assessment and at-home care adjustment.

Baseline characteristics, including sex, age, department, length of hospital stay (LOS), baseline NRS, baseline PSQI, baseline constipation, and baseline analgesic use, were collected for comparison between the two groups.

#### 2.1.3 Control-group protocol

Patients in the control group received routine post-discharge telephone follow-up. Telephone follow-up was conducted once weekly for four consecutive weeks after discharge. During each call, nurses asked about general symptom status, medication adherence, pain status, and adverse reactions. Patients were advised to seek medical attention when uncontrolled pain, severe constipation, or other severe adverse symptoms occurred. The control group did not receive WeChat-based daily monitoring, 24-hour online support, scheduled home visits, or structured continuity-of-care nursing activities.

#### 2.1.4 Nursing intervention protocol (experimental group)

##### 2.1.4.1 Establishment of a continuity-of-care nursing team and discharge preparation

In this study, a continuity-of-care nursing team was established to provide better care for the patients. The team comprised one head nurse from the Department of Medical Oncology, one traditional Chinese medicine (TCM)-specialized nurse, and four oncology specialist nurses. All team members received standardized training and passed an assessment on cancer pain management prior to study commencement. The training covered basic knowledge of cancer pain, assessment methods, principles of analgesic treatment, pain nursing and communication skills, and observation and nursing care for complications as well as adverse drug reactions (ADRs).

Before discharge, an individualized patient file was created and one-to-one guidance was provided to patients and family caregivers. Medication guidance for home-based pain management was delivered, including the purpose and potential adverse effects of each analgesic. Patients and caregivers were instructed to record daily pain intensity at home using the NRS, and were told to contact the team members via telephone or WeChat when NRS ≥ 4 or when new symptoms occurred.

In addition, patients were instructed in non-pharmacological pain management approaches:

###### 2.1.4.1.1 Music therapy

In the intervention group, music therapy was performed twice a day at 09:00 and 15:00. Patients were informed to keep the listening environment clean, quiet, dimly lit, and maintained at appropriate temperature and humidity, to wear loose clothing, and to sit or lie on their back comfortably. Slow, soft music was delivered via electronic devices, and the volume was adjusted to a comfortable level. The participants were told to close their eyes, relax their muscles, and actively listen for 30 minutes per session, two times a day.

###### 2.1.4.1.2 Distraction techniques

In the intervention group, the nurses were also instructed to communicate with patients to understand their family situation, cultural background, pain severity, and interests, and provided feasible distraction skills such as deep breathing, watching television, reading, talking, and simple handicrafts.

###### 2.1.4.1.3 TCM acupressure at Neiguan (Pericardium 6, PC6)

In addition to psychosocial strategies, physiological-level care was provided through TCM acupressure. Under the guidance of the TCM-specialized nurse, patients were instructed to adopt a supine position and apply bilateral digital pressure at Neiguan (PC6), located on the palmar aspect of the forearm approximately two finger-widths proximal to the wrist crease, between the tendons of the palmaris longus and flexor carpi radialis. Pressure was gradually increased from light to moderate until the patient reported local sensations such as distension, numbness, soreness, or mild pain. The intervention was performed twice daily for 20 minutes per session. To prevent skin irritation or injury, abrasive rubbing, kneading, or vigorous manipulation of the acupoint was avoided. Patients were also advised to keep the skin clean and intact to reduce the risk of infection. After discharge, patients and caregivers were instructed on how to perform the procedure at home.

##### 2.1.4.2 Participation in hospital-based pain management activities

Patients were encouraged to participate in pain management activities organized by the hospital. Illustrated pain management booklets were distributed. Patients with good self-management were invited to serve as role models to share self-care experiences and demonstrate key precautions in daily life.

##### 2.1.4.3 WeChat-based follow-up and monitoring

Before discharge, a dedicated WeChat group was created and patients and their family caregivers were invited to join. By joining the group, patients could report their home status and communicate with the team. In addition, team members could provide timely responses and supervision to promote self-care regarding daily living, medication adherence, psychological adjustment, and diet. Health education materials were regularly posted in the group to encourage proactive communication and timely reporting of discomfort. Knowledge related to cancer pain was posted weekly using images or videos.

In addition, online video communication was performed once a week to inquire about the patients’ pain and sleep status at home, and to monitor for potential adverse drug reactions such as nausea or vomiting, constipation, dizziness or headache, and respiratory depression. Should any of the above occur, or if pain control was not satisfactory, the patient was immediately advised to contact the medical staff and seek immediate medical attention at the hospital.

##### 2.1.4.4 Telephone follow-up

Telephone follow-up was conducted once weekly for four consecutive weeks after discharge, scheduled to avoid the patients’ resting hours. Follow-up content included NRS score for pain control, occurrence of breakthrough pain, medication adherence, incidence of adverse drug reactions, gastrointestinal symptoms, diet and sleep, and psychological status. Questions raised by patients were addressed during the calls. If abnormalities were identified, patients were advised to seek medical attention promptly, and the frequency of subsequent follow-up was adjusted accordingly.

##### 2.1.4.5 Home visit

One home visit was performed by an oncology specialist nurse two weeks after discharge. The visit assessed home-based care status, including physical examination, pain assessment, medication adherence, and individualized care plan adjustment. Evidence-based guidance was provided on managing constipation and improving sleep quality, as well as health education related to pain for patients and family caregivers during the visit. Barriers to healthy behaviors were identified, and tailored support was provided to help patients overcome them. The nurse affirmed the patient’s positive self-management behaviors that the patient was already undertaking, and encouraged them to continue with these.

##### 2.1.4.6 Post-follow-up documentation

After each follow-up, patient-specific information was summarized and discussed within the team, and all follow-up records were documented in the individualized patient file.

### 2.2 Observation indicators

Assessment of the indicators were conducted at discharge as baseline and 4 weeks after discharge as follow-up endpoint.

#### 2.2.1 Primary outcome cancer pain control

Two indicators were used for cancer pain control. The first was Pain NRS score, a single-item scale with 0 points representing no pain and 10 points representing the worst imaginable pain. The second was pain control satisfaction, which was categorized as satisfied (pain well-controlled, daily activities unaffected), general (pain partially relieved but tolerable) or dissatisfied (pain poorly controlled or affecting sleep and activities) based on patient self-report.

The operational definitions of pain control satisfaction categories are provided in **Supplementary Table 1**.

#### 2.2.2 Adverse reactions as the secondary outcome

Three indicators were used for adverse reactions. The first was constipation, a binary indicator recorded as Yes or No. Constipation was defined as fewer than three bowel movements per week and/or constipation-related symptoms such as hard or lumpy stools, straining, or difficulty in defecation. The second was analgesic-related adverse reactions, a binary indicator recorded as Yes or No. It included opioid-related side effects such as nausea, dizziness and respiratory depression, and non-opioid side effects such as gastrointestinal irritation from indomethacin suppositories. It was extracted from the “Adverse Reactions” field. The third was analgesic use, recorded as Yes or No from the “Analgesic Use” field. Specific drug types such as oxycodone, fentanyl transdermal patch and tramadol were obtained from the “Current Analgesic Name” field to adjust for confounding by medication type.

#### 2.2.3 Quality of life as the secondary outcome

PSQI was used to assess quality of life. It is a validated 19-item scale evaluating sleep quality over the past month, with total scores ranging from 0 to 21. A score of 0 represents excellent sleep and 21 represents poor sleep. According to clinical standards, scores >7 were defined as poor sleep quality.

A PSQI score >7 was used to define poor sleep quality in this study because a cut-off score of 8 has been used in studies involving Chinese populations (13). Although a cut-off of >5 is commonly recommended in the original PSQI scoring and has also been reported in Chinese cancer-related populations, the present study analyzed PSQI mainly as a continuous outcome, and the categorical classification was used only as a supportive descriptive analysis (14).

### 2.3 Data collection and quality control

#### 2.3.1 Data sources

Data were collected from two sources. Baseline information was extracted from the Electronic Medical Records (EMRs) at discharge, including demographic information in the form of “Gender” and “Age”, clinical information such as “Diagnosis,” “Department,” “Admission Time,” “Discharge Time,” and “Length of Hospital Stay,” and baseline symptom scores in the form of “Pain NRS Score” and “PSQI Score.”

Data on follow-up were available for all the subjects by phone, WeChat, or home visits and supplemented by verification from medical and community records to assure completeness and accuracy of information.

#### 2.3.2 Quality control

Quality control was implemented in three aspects. First, standardized training on symptom assessment, data collection, and follow-up procedures was conducted for all nurses to minimize study-wide variations. Second, dual verification was conducted: two independent researchers reviewed all data twice and compared them with the EMRs or follow-up documents, and any inconsistencies were resolved by consulting the original documents. Third, data consistency checking was performed before statistical analysis to ensure that baseline, follow-up, and change-score variables were internally consistent. NRS and PSQI were patient-reported instruments collected using standardized questionnaires; therefore, inter-rater reliability assessment such as kappa statistics was not applicable.

### 2.4 Statistical analysis

Continuous outcomes were analyzed using between-group comparisons at baseline and 4 weeks, as well as change scores calculated as the 4-week value minus the baseline value. Pearson correlation was used to examine linear associations between continuous NRS and PSQI scores. Because NRS and PSQI are score-based patient-reported measures and may not fully satisfy normality assumptions, Spearman correlation was additionally used as a non-parametric sensitivity analysis to assess the robustness of the correlation findings. Partial Pearson correlation was used to assess the association between NRS and PSQI after controlling for group assignment.

To address baseline imbalance in analgesic use, adjusted linear regression models equivalent to ANCOVA were performed for NRS and PSQI outcomes. Group was entered as the main independent variable, and baseline outcome values, baseline analgesic use, and baseline constipation were entered as covariates. For constipation, a sensitivity analysis was performed among participants who had constipation at baseline.

*Post-hoc* power analysis was performed for the main NRS and PSQI outcomes using the observed between-group differences and a two-sided α of 0.05. The results are provided in **Supplementary Table 2**.

## 3 Results

### 3.1 Baseline characteristics of participants

The baseline characteristics of 136 participants are shown in **Table 1**. Most baseline characteristics were comparable between the two groups, including sex, age, length of hospital stay, department, baseline NRS, baseline PSQI, and baseline constipation. Baseline NRS was 4.87 ± 0.64 in the control group and 4.75 ± 0.66 in the experimental group (P = 0.293). Baseline PSQI was 12.66 ± 1.30 and 12.47 ± 1.17, respectively (P = 0.384). Baseline constipation was also comparable between groups (P = 0.207). Baseline analgesic use differed between groups (100.0% vs. 72.1%, P < 0.001), and was therefore included in adjusted analyses.

**Table 1:** Baseline demographic and clinical characteristics of participants.

Characteristic	Control (n=68)	Experimental (n=68)	P value
Male, n (%)	40 (58.8%)	46 (67.6%)	0.286
Female, n (%)	28 (41.2%)	22 (32.4%)	
Age, mean ± SD, years	65.03 ± 8.96	67.57 ± 8.56	0.093
Length of stay, mean ± SD, days	11.57 ± 7.92	12.34 ± 8.76	0.594
Baseline NRS, mean ± SD	4.87 ± 0.64	4.75 ± 0.66	0.293
Baseline PSQI, mean ± SD	12.66 ± 1.30	12.47 ± 1.17	0.384
Baseline constipation, yes, n (%)	48 (70.6%)	41 (60.3%)	0.207
Baseline constipation, no, n (%)	20 (29.4%)	27 (39.7%)	
Baseline analgesic use, yes, n (%)	68 (100.0%)	49 (72.1%)	<0.001
Baseline analgesic use, no, n (%)	0 (0.0%)	19 (27.9%)	
Medical oncology, n (%)	50 (73.5%)	52 (76.5%)	0.692
Department of Tumor Radiotherapy, n (%)	18 (26.5%)	16 (23.5%)	

### 3.2 Outcomes of cancer pain control

Pain control outcomes are presented in **Table 2**. Baseline NRS scores were comparable between the two groups (P = 0.293). At 4 weeks, the experimental group had a significantly lower NRS score than the control group (1.46 ± 0.50 compared with 2.93 ± 0.31, P < 0.001). The reduction in NRS from baseline to 4 weeks was also greater in the experimental group than in the control group (-3.29 ± 0.79 compared with -1.94 ± 0.73, P < 0.001). Pain control satisfaction differed significantly between groups (P < 0.001), with a higher proportion of satisfied patients in the experimental group.

**Table 2:** Comparison of pain NRS score and pain control satisfaction between control and experimental groups during the 4-week post-discharge follow-up.

Outcome	Control (n=68)	Experimental (n=68)	P value
Baseline NRS, mean ± SD	4.87 ± 0.64	4.75 ± 0.66	0.293
4-week NRS, mean ± SD	2.93 ± 0.31	1.46 ± 0.50	<0.001
ΔNRS, mean ± SD	-1.94 ± 0.73	-3.29 ± 0.79	<0.001
Pain control satisfaction, Satisfied, n (%)	29 (42.6%)	65 (95.6%)	<0.001
Pain control satisfaction, Fair, n (%)	22 (32.4%)	3 (4.4%)	
Pain control satisfaction, Dissatisfied, n (%)	17 (25.0%)	0 (0.0%)	

ΔNRS was calculated as the 4-week NRS minus the baseline NRS; negative values indicate improvement.

### 3.3 Outcomes of adverse reactions

Constipation and adverse reactions are shown in **Table 3**. Baseline constipation was comparable between the control and experimental groups (70.6% compared with 60.3%, P = 0.207). At 4 weeks, constipation was significantly less frequent in the experimental group than in the control group (38.2% compared with 72.1%, P < 0.001).

**Table 3:** Constipation and other adverse drug reactions during the 4-week follow-up.

Outcome	Control (n=68)	Experimental (n=68)	P value
Baseline constipation, yes, n (%)	48 (70.6%)	41 (60.3%)	0.207
4-week constipation, yes, n (%)	49 (72.1%)	26 (38.2%)	<0.001
Other ADRs at 4 weeks, yes, n (%)	17 (25.0%)	18 (26.5%)	0.844
Sensitivity among baseline-constipated patients: 4-week constipation, yes, n (%)	42/48 (87.5%)	26/41 (63.4%)	0.008

Among participants who had constipation at baseline, 4-week constipation remained less frequent in the experimental group than in the control group (63.4% compared with 87.5%, P = 0.008). Other adverse drug reactions did not differ significantly between groups (P = 0.844).

### 3.4 Outcomes of quality of life

PSQI outcomes are shown in **Table 4**. Baseline PSQI scores were comparable between the control and experimental groups (P = 0.384). At 4 weeks, the experimental group had a significantly lower PSQI score than the control group (5.93 ± 1.13 vs. 11.77 ± 1.60, P < 0.001). The reduction in PSQI from baseline to 4 weeks was also greater in the experimental group (-6.54 ± 1.08 vs. -0.88 ± 1.16, P < 0.001).

**Table 4:** PSQI score and 4-week sleep-quality classification.

Outcome	Control (n=68)	Experimental (n=68)	P value
Baseline PSQI, mean ± SD	12.66 ± 1.30	12.47 ± 1.17	0.384
4-week PSQI, mean ± SD	11.77 ± 1.60	5.93 ± 1.13	<0.001
ΔPSQI, mean ± SD	-0.88 ± 1.16	-6.54 ± 1.08	<0.001
4-week good sleep quality (PSQI ≤7), n (%)	0 (0.0%)	56 (82.4%)	<0.001

ΔPSQI was calculated as the 4-week PSQI minus the baseline PSQI; negative values indicate improvement.

At baseline, all participants in both groups were classified as poor sleepers. At 4 weeks, 56 patients (82.4%) in the experimental group had good sleep quality, whereas all patients in the control group remained poor sleepers (P < 0.001).

### 3.5 Cancer pain and quality of life association

Correlation analyses between pain intensity and sleep quality are summarized in **Table 5**. Pearson correlation was used to examine the association between 4-week NRS and PSQI, and Spearman correlation was used as a sensitivity analysis. In the overall sample, 4-week NRS was positively correlated with 4-week PSQI using both Pearson correlation (r = 0.785, P < 0.001) and Spearman correlation (ρ = 0.769, P < 0.001). However, after controlling for group, the partial Pearson correlation was no longer significant (r = -0.013, P = 0.877), suggesting that the unadjusted association was largely explained by group differences.

**Table 5:** Correlation analyses between pain intensity and sleep quality.

Analytical scenario	Coefficient (r/ρ)	N	df	P value
Pearson correlation between 4-week NRS and PSQI	0.785	136	—	<0.001
Spearman correlation between 4-week NRS and PSQI	0.769	136	—	<0.001
Partial Pearson correlation between 4-week NRS and PSQI, controlling for group	-0.013	136	133	0.877
Pearson correlation between ΔNRS and ΔPSQI	0.671	136	—	<0.001
Partial Pearson correlation between ΔNRS and ΔPSQI, controlling for group	0.187	136	133	0.030

Changes in NRS and PSQI were also positively correlated (r = 0.671, P < 0.001). After controlling for group, this association was attenuated but remained statistically significant (r = 0.187, P = 0.030).

### 3.6 Adjusted and sensitivity analyses

Adjusted and sensitivity analyses are shown in **Table 6**. After adjustment for baseline outcome values, baseline analgesic use, and baseline constipation, the group effect remained significant for both NRS and PSQI outcomes. The experimental group had lower 4-week NRS and PSQI scores and greater reductions in both outcomes after adjustment. In the sensitivity analysis restricted to participants with baseline constipation, the experimental group had lower odds of 4-week constipation than the control group (OR = 0.248, 95% CI: 0.085 to 0.719, P = 0.008).

**Table 6:** Adjusted and sensitivity analyses.

Model	Group effect	95% CI	P value	R2
NRS at 4 weeks adjusted for baseline NRS, baseline analgesic use, and baseline constipation	-1.409	-1.564 to -1.253	<0.001	0.765
Change in NRS adjusted for baseline NRS, baseline analgesic use, and baseline constipation	-1.409	-1.564 to -1.253	<0.001	0.838
PSQI at 4 weeks adjusted for baseline PSQI, baseline analgesic use, and baseline constipation	-5.760	-6.157 to -5.363	<0.001	0.896
Change in PSQI adjusted for baseline PSQI, baseline analgesic use, and baseline constipation	-5.762	-6.159 to -5.365	<0.001	0.882
Sensitivity among baseline-constipated patients: OR for 4-week constipation, Experimental vs Control	OR = 0.248	0.085 to 0.719	0.008	—

## 4 Discussion

This randomized study enrolled 136 patients with digestive tract tumors after discharge and evaluated the benefits of multi-channel follow-up as a continuous nursing strategy. Our study provides clinical evidence on post-discharge pain management, adverse reactions, constipation, and sleep quality in this population. The intervention might be associated with positive outcomes in terms of cancer pain management, adverse effects, sleep satisfaction, as well as the pain–sleep relationship, indicating both symptom-specific and general improvements.

The experimental group showed a significant decrease of pain intensity, and the mean scores of NRS were significantly lower than those of the control group (1.46± 0.50 vs. 2.93± 0.31), and the satisfaction rate was significantly higher (95.6% vs. 42.6%). These findings suggest that multi-channel follow-up may improve post-discharge pain control in routine practice, where inadequate cancer pain management remains common (15, 16). The strength of the multi-channel approach is its integration of continuity and personalization: it included nurse-led telephone assessments, real-time WeChat-based support, and home visits to facilitate prompt analgesic modification and tailored symptom management. Previous research has demonstrated the effectiveness of nurse-led telehealth programs in reducing pain severity (9, 12, 17). Our study contributes to this body of evidence by concentrating on patients with digestive tract tumors, a population facing distinctive post-discharge obstacles, including gastrointestinal motility disorders and rapidly changing pain. The alignment of the lower NRS score as the objective outcome, with the greater satisfaction as the subjective outcome, underscores the holistic impact of the intervention.

With respect to adverse reactions, the intervention significantly reduced the incidence of constipation (38.2% vs. 72.1%). This finding remained consistent among participants who had constipation at baseline. This is clinically meaningful, as constipation is a frequent and burdensome problem in patients with digestive tract tumors, compounded by both tumor-related factors and analgesic use (18, 19). Our intervention for constipation was based on evidence-based non-pharmacologic approaches, including recommendations concerning diet, hydration, and physical activity given at follow-up. Conversely, other analgesic-related side effects such as nausea and dizziness did not differ significantly between the two groups, in line with their status as more medication-dependent and less likely to be influenced by nursing interventions. Importantly, the intervention was not associated with an increased risk of adverse reactions, which confirmed its safety. These findings add to the benefits of nursing-delivered supportive care administered in combination with pharmacologic treatments. Because the intervention combined telephone follow-up, WeChat-based monitoring, home visits, medication guidance, dietary and hydration advice, and activity-related supportive care, the observed constipation benefit should be interpreted as the integrated effect of multi-channel follow-up and constipation-oriented supportive care rather than the independent effect of any single component.

Sleep quality as evaluated by PSQI was greatly improved in the experimental group, 56 patients (82.4%) in the experimental group achieved good sleep quality at 4 weeks. This trend represents a change from chronic sleep deprivation to restorative sleep with potential impact not only on energy and cognitive function during the day but also on compliance with regimens for recovery. Prior randomized trials have suggested that structured follow-up improves QoL among the cancer patients (20, 21), but our study provides disease-specific evidence and highlights that multi-channel follow-up enhances sleep, through the pathways of both pain reduction and sleep hygiene guidance.

Pain intensity and sleep quality improved in parallel in the experimental group. This finding is clinically plausible because reduced pain may directly contribute to better sleep. In the overall sample, 4-week NRS was positively correlated with PSQI, indicating that patients with higher pain intensity tended to report poorer sleep quality. After controlling for group assignment, this cross-sectional association became non-significant, suggesting that the unadjusted association was largely driven by between-group differences in intervention exposure and symptom improvement (2, 22). The experimental group experienced lower pain intensity, which was associated with better sleep quality.

Taken together, our findings contribute three key insights. First, while prior reviews have shown the overall effectiveness of nurse-led remote follow-up interventions for cancer patients, our study provides disease-specific evidence for patients with digestive tract tumors. Second, unlike prior studies that primarily addressed constipation through pharmacologic means, we highlight the efficacy of non-pharmacologic nursing strategies. Third, our study showed that the association between pain intensity and sleep disturbance was attenuated after accounting for group effects, providing a more cautious interpretation of the relationship between pain control and sleep improvement in post-discharge care.

Strengths of this study include its prospective controlled design, randomization, use of validated assessment tools such as NRS and PSQI scales, rigorous data quality procedures, and statistical adjustment for baseline differences in analgesic use. Nonetheless, there are several limitations to mention. First, this was a single-institution study, which may limit the generalizability of the findings to community hospitals or patients with more advanced metastatic disease. Second, the follow-up duration was restricted to 4 weeks; therefore, long-term outcomes such as survival, emotional well-being, role functioning, and sustained symptom control were not assessed. Third, although baseline analgesic use was statistically adjusted in the multivariable models, a comparative subgroup analysis among patients not using analgesics at baseline could not be performed because all patients in the control group used analgesics. Fourth, the nature of the intervention prevented blinding of patients and researchers, and the primary outcomes, including NRS and PSQI, were based on patient-reported data; therefore, the possibility of detection or reporting bias cannot be fully excluded. Fifth, although a *post-hoc* power analysis was added during revision, the original target sample size was determined based on feasibility and expected patient flow rather than an *a priori* statistical sample-size calculation. Finally, changes in analgesic doses during follow-up were not fully documented, and other potential confounders, such as comorbidities and differences in treatment regimens, were not completely collected or adjusted for.

The implementation of multi-channel follow-up also requires consideration of resource feasibility. Compared with routine telephone follow-up, this model requires trained oncology nurses, structured documentation, digital communication tools, and time allocated for WeChat-based monitoring and home visits. Therefore, the model may be more feasible when applied to high-risk patients with uncontrolled pain, poor medication adherence, severe constipation, or limited family support. In routine practice, a stepwise strategy may be adopted, with telephone follow-up as the basic component and WeChat monitoring or home visits added according to patient risk and available nursing resources.

The results of the present study imply, from a clinical perspective, that multi-channel follow-up as continuous nursing consisting of telephone assessment, digital communication, and home visits for patients with digestive tract tumors should be included in the discharge care for this group of patients. Tertiary prevention or nursing interventions need to focus the modifiable adverse effects, such as constipation and not just on the side effects of the medication. Clinical practice guidelines may also give greater attention to the co-occurrence of pain and sleep disturbance, while recognizing that the relationship between these symptoms should be interpreted cautiously and requires further investigation in prospective multicenter studies. It is expected that future research in the form of multi-center trials with longer follow-up evaluating extended QoL and subgroup analyses to identify potential differential benefits for elderly patients and those receiving high-dose opioids should be conducted.

## 5 Conclusion

In summary, multi-channel follow-up as continuity-of-care nursing improved pain control, reduced constipation, and enhanced sleep quality among post-discharge patients with digestive tract tumors. The observed association between pain intensity and sleep quality was attenuated after adjustment for group assignment, but this finding should be interpreted as associative rather than causal. Collectively, these results offer strong support for the translation of this model into clinical practice, filling critical post-discharge care gaps and improving patient-centered outcomes for this high-risk population.

## Data Availability

The raw data supporting the conclusions of this article will be made available by the authors, without undue reservation.
