# Supplementary material for: Impact of multi-channel follow-up as continuous nursing on cancer pain control, adverse reactions, and quality of life in patients with digestive tract tumors: a controlled study of 136 cases
Source: Front Oncol. 2026 Jul 20;16:1868504. doi: 10.3389/fonc.2026.1868504 (PMC13429404; doi:10.3389/fonc.2026.1868504)
Supplement: Supplementary file 1 [file Table1.docx]

**Table S1.** Single-item assessment of pain control satisfaction.

| **Category** | **Operational definition** |
| --- | --- |
| Satisfied | Pain was well controlled and did not affect daily activities or sleep. |
| Fair | Pain was partially relieved and generally tolerable, but occasional discomfort remained. |
| Dissatisfied | Pain was poorly controlled or affected sleep and daily activities. |
