# Supplementary material for: Impact of multi-channel follow-up as continuous nursing on cancer pain control, adverse reactions, and quality of life in patients with digestive tract tumors: a controlled study of 136 cases
Source: Front Oncol. 2026 Jul 20;16:1868504. doi: 10.3389/fonc.2026.1868504 (PMC13429404; doi:10.3389/fonc.2026.1868504)
Supplement: Supplementary file 2 [file Table2.docx]

**Table S2.** Post-hoc power analysis.

| **Outcome** | **Cohen’s d** | **Post-hoc power** |
| --- | --- | --- |
| 4-week NRS | -3.51 | 1 |
| 4-week PSQI | -4.21 | 1 |
| Change in NRS | -1.77 | 1 |
| Change in PSQI | -5.04 | 1 |
